# The sensory channel of presentation alters subjective ratings and autonomic responses toward disgusting stimuli—Blood pressure, heart rate and skin conductance in response to visual, auditory, haptic and olfactory presented disgusting stimuli

**DOI:** 10.3389/fnhum.2013.00510

**Published:** 2013-09-03

**Authors:** Ilona Croy, Kerstin Laqua, Frank Süß, Peter Joraschky, Tjalf Ziemssen, Thomas Hummel

**Affiliations:** ^1^Department of Otorhinolaryngology, Smell and Taste Clinic, University of Dresden Medical SchoolDresden, Germany; ^2^Department of Psychosomatic Medicine, University of Dresden Medical SchoolDresden, Germany; ^3^Department of Occupational and Social Medicine, University of Dresden Medical SchoolDresden, Germany; ^4^Center of Clinical Neuroscience, Neurological University Clinic, University of Dresden Medical SchoolDresden, Germany

**Keywords:** disgust, olfaction, vision, audition, touch, rating, heart rate, blood pressure

## Abstract

Disgust causes specific reaction patterns, observable in mimic responses and body reactions. Most research on disgust deals with visual stimuli. However, pictures may cause another disgust experience than sounds, odors, or tactile stimuli. Therefore, disgust experience evoked by four different sensory channels was compared. A total of 119 participants received 3 different disgusting and one control stimulus, each presented through the visual, auditory, tactile, and olfactory channel. Ratings of evoked disgust as well as responses of the autonomic nervous system (heart rate, skin conductance level, systolic blood pressure) were recorded and the effect of stimulus labeling and of repeated presentation was analyzed. Ratings suggested that disgust could be evoked through all senses; they were highest for visual stimuli. However, autonomic reaction toward disgusting stimuli differed according to the channel of presentation. In contrast to the other, olfactory disgust stimuli provoked a strong decrease of systolic blood pressure. Additionally, labeling enhanced disgust ratings and autonomic reaction for olfactory and tactile, but not for visual and auditory stimuli. Repeated presentation indicated that participant's disgust rating diminishes to all but olfactory disgust stimuli. Taken together we argue that the sensory channel through which a disgust reaction is evoked matters.

## Introduction

Disgust is ranked among the basic emotions of humans. One of the most popular theories states that there are six inborn basic emotions in human, which are present across all cultures. These are happiness, anger, disgust, sadness, fear, and surprise (Ekman et al., 1983). Although it has been questioned if emotions can be categorized in such a way [see Barrett et al. (2007) and Panksepp (2007) for ongoing debate], it is undisputed that all healthy persons are able to feel disgust [for overview see Rozin et al. (2000) and Tybur et al. (2009)].

Emotions can be evoked by environmental cues and visual or visual-auditory material often serves as emotional trigger in an experiment [for overview, see for instance Kreibig (2010)]. Coming from olfactory research, we observed that odorous cues have a high potential to evoke disgust. It might even be one of the key functions of the olfactory system to warn about microbial threats by evoking disgust (Stevenson, 2010). There are some studies indicating that although not every emotion can be induced easily using odors, disgust can be evoked reliably by the sense of smell (Alaoui-Ismaili et al., 1997b; Bensafi et al., 2002; Croy et al., 2011).

Based on this, we wondered if the sensory channel of presentation contributes to emotional experience. Darwin already noted 150 years ago that different senses may have a special relation toward disgust. He defined disgust as “something revolting, primary in relation to the senses of taste and smell, as actually perceived or vividly imagined; and secondarily to anything which causes a similar feeling, through the sense of smell, touch, and even eyesight” (Darwin, 1872). Nevertheless, most research on disgust deals with pictures or videos [for overview see Kreibig (2010)].

Emotional stimuli are processed in two stages: First persons orient to the sensory input and process the contextual details. Heart rate (HR) decelerates and skin conductance (SCL) decreases mirroring the parasympathetic-sympathetic co-activated orienting reaction. Then the relevant information is retrieved from memory and the participants implicitly prepare for relevant action (Bradley et al., 2001). For threatening stimuli, orienting is normally followed by a sympathetic driven increase of HR preparing the body for the fight- or flight reaction (Bradley et al., 2001). Disgusting stimuli however might have other behavioral requirements. The typical disgust elicitors are spoiled food, illness-related stimuli and feces (Rozin and Fallon, 1987; Rozin et al., 2000; Vaitl et al., 2005). Instead of a fast and sympathetic dominated typical fight- or flight reaction another behavior seems reasonable: Going away from the source or removing the source from the body, for instance by vomiting. In fact, disgust causes specific reaction patterns, observable in mimics and typical body reaction up to regurgitation (Rozin and Fallon, 1987) and is accompanied by an increase of skin conductance level and a decrease of heart rate (Vaitl et al., 2005).

Why should disgust reaction differ according to the evoking sensory channel? The senses fulfill different functions, have different neurological pathways and access to explicit memory differs between the senses. Based on those considerations, we hypothesize that disgust response differs depending on the sensory channel of disgust perception.

The main function of disgust is avoidance of disease (Oaten et al., 2009). Therefore, disgust motivates rejection of potential health threatening objects especially from microbial sources, such as found in wounds, spoiled food and organic waste. Typical disgust objects seem to be in near distance: Most microbial threats have to be touched, inhaled or eaten to infiltrate the body powerfully. Consequently, we would expect that proximal senses, such as touch and olfaction can evoke strong disgust and produce enhanced reaction compared to stimuli processed through the sense of vision or audition.

Second, the senses use different neurological pathways. Specialized receptors of each sensory channel transform environmental inputs into electrical signals, which are transported to the related primary sensory cortex. In contrast to other senses, the olfactory system projects ipsilaterally and most fibers bypass the thalamus and project directly into amygdala, piriform cortex, and entorhinal cortex (Gottfried, 2006).

Third, although emotions can occur independently from cognition (Izard, 1992), it is undisputed that cognition influences emotional experience. Because of the organization of working memory, visual and verbal cues can be identified easily (Baddeley and Hitch, 1974). Environmental stimuli processed through the visual and auditory channel may therefore trigger much contextual information. This helps in selecting the appropriate behavioral response. However, odors are not that easy to identify (Jonsson and Olsson, 2003). Accordingly, labeling has a strong influence on emotional rating of odors. For example, participants liked the very same odor significantly less and even processed it in a different way, when it was labeled “body odor” instead of “cheddar cheese” (De Araujo et al., 2005). We hypothesize that a label enhances disgust perception for olfactory, but not for visual and auditory stimuli.

Related to the assumptions above, objects presented by different channels might have a differential potential to stay in memory. If the same disgusting object is presented repeatedly, the reaction to this object might change according to the sensory channel. We argued that verbal and visual stimuli are easy to categorize, which enhances recognition in repeated presentation. A study conducted on aversive (fearful) stimuli for instance showed that autonomic response to aversive pictures decreased after some days (Tabibnia et al., 2008). For non-verbal auditory, olfactory, and tactile stimuli categorization and therefore recognition may be more difficult. This may lead to slower habituation of emotional response in case of repeated presentation.

Taken together we have reason to assume that the sensory channel of presentation contributes to disgust reactions. Former studies indicate that disgust can be evoked through the olfactory (e.g., Bensafi et al., 2002), tactile (e.g., Hertenstein et al., 2009; Oum et al., 2011), and visual (e.g., Collet et al., 1997) channel as well as through a combination of the visual and auditory channels using film-clips (e.g., Kunzmann and Gruhn, 2005). However, whether disgust perception differs with regard to the sensory channel has—to the best of our knowledge—not been studied yet.

We attempted to systematically evaluate disgust reactions evoked by the visual, auditory, tactile, and olfactory sense^1^. Stimuli of the three most pronounced disgust categories were presented through each of the sensory channels and subjective perception and reactivity of the autonomic nervous system was analyzed. Previous studies examining autonomic reactivity for disgusting stimuli found differences in facial electromyographic activity (i.e., Bensafi et al., 2002), skin potential (i.e., Alaoui-Ismaili et al., 1997a; Collet et al., 1997), systolic blood pressure (i.e., Prkachin et al., 1999; Kunzmann and Gruhn, 2005), and heart rate (i.e., Alaoui-Ismaili et al., 1997a; Rohrmann et al., 2009), for overview see Kreibig (2010). We concentrated on measurements of skin conductance, systolic blood pressure, and heart rate. In order to minimize a potential bias in stimulus selection for single stimuli, results of all three disgust categories were averaged for each sensory channel. To test the influence of cognition, semantic information was added to one third of the stimuli. The experiment was repeated twice with a subgroup of the same participants.

## Materials and methods

### Participants

A total of 124 healthy people participated in the study, 5 had to be excluded from analysis because of technical problems with the autonomic measurement. Thus, data is analyzed from 119 participants (60 women, 59 men, age range 18–36 years, mean = 22.7 years; standard deviation = 3 years). Most of them were graduate students of the Technical University of Dresden. Completion of a detailed medical history form by each participant enabled confirmation of his or her good physical health. Normal olfactory function was ascertained in all participants using an olfactory screening test (Hummel et al., 2001, 2007).

In order to analyze the influence of repeated presentation, 43 sex matched participants—equally spread around the labeling condition (see below)—took part in two repetitions of the experiment. The study followed the Declaration of Helsinki on Biomedical Research Involving Human Subjects and was approved by the Ethics Committee from the University of Dresden Medical School. All participants provided written informed consent. They received a small amount of money for their participation.

### Materials

Disgust and control stimuli were presented through the visual, auditory, tactile, and olfactory channel. To enhance the validity of the experiment, disgusting stimuli of three different categories were chosen for each sensory channel: “spoiled food,” “illness related,” and “feces.” Choice of categories was based on the current literature (Rozin and Fallon, 1987; Rozin et al., 2000; Vaitl et al., 2005). The study did not aim to compare different disgust categories, but different categories were presented to enhance the validity of the experiment and merged for analysis. For control purpose, stimuli with low emotional value were used. The sound of a person writing from the International Affective Digital Sounds database (358) was selected and accordingly a picture of a pencil was used for visual stimulation and pencil for tactile stimulation. As there is no matching odor, we decided to use chocolate odor [Bell flavors and Fragrances (diluted up to 66% in polypropylenglycol)], which is—based on previous experience in our laboratory—perceived as rather neutral.

A description of the stimuli is presented in Table 1. The order of the 16 different presentations in total was pseudo-randomized across participants. Each participant received one disgust category labeled while the other two were presented without the participants' knowing what the stimulus was supposed to be. For instance for participant A all “spoiled food” stimuli had a label, when presented through the four channels. But none of the “illness related” or “feces” stimuli was presented labeled. Order of labeling was randomized across participants. In case of repeated presentation the same stimuli were labeled for each participant.

**Table 1:** **Description of disgust and control stimuli**.

	**Vision**	**Audition**	**Touch**	**Olfaction**
Spoiled food	Picture of spoiled pasta^*^	Sound of a woman vomiting^*^	Rubber pasta^*^	Carbon disulfide
Illness related	Picture of melanom at the toe	Sound of a women coughing	Tissue smeared with soap^*^	Artificial sweat
Feces	Picture of feculent toilet	Sound of a person with diarrhea^*^	Feces made of flour and water^*^	Odor of feces
Control	Picture of a pencil^*^	Sound of a person writing	Pencil^*^	Odor of chocolate

Olfactory stimuli are diluted in polypropylenglycol. In detail: picture of melanom taken from Duale Reihe Dermatologie, by courtesy of Thieme Verlag, picture of feculent toilet taken from the International Affective Picture System (IAPS, 9301); sound of coughing women International Affective Digital Sounds (IADS, 242), sound of a person writing IADS (358); the following odors were used: Civette Base 847 (Fragrance Resources, Hamburg, Germany diluted up to 5% in polypropylenglycol), carbon disulfide (order # 180173; Sigma, Deisenhofen, Germany), artificial sweat (2-methyl-3-mercapto-butanol; Unilever, Port Sunlight, UK), Chocolade odor, Bell flavors and Fragrances,(diluted up to 66% in polypropylenglycol). All items marked with ^*^ were made by the experimenters.

### Ratings

Ratings for *arousing and hedonic qualities* of the stimuli was assessed using the Self Assessment Manikins (Lang, 1980), a visual analog rating scale which prompts responses for arousal and valence ratings on a 9-point-scale, whereby “1” means “not at all” pleasant or arousing and “9” means “extremely” pleasant or arousing.

To judge which *emotion* was evoked by the stimuli, participants were asked to state to which degree the presented stimulus evoked the following five basic emotions: happiness, disgust, anger, anxiety, and sadness. Although listed as one of the basic emotions, we decided not to analyze “surprise.” Among the proposed basic emotions by Ekman et al. (1983), surprise is discussed most controversial and might rather reflect orienting reaction (Posner et al., 2005). Ratings were given on an analog rating scale from 1 to 9 (the emotion is experienced “not at all” to “extremely strong”).

### Autonomic measurements

Recordings were performed in a sitting position in an air-conditioned laboratory. Food intake and consumption of caffeine or nicotine had to be stopped at least 1 h before the examination. Continuous monitoring of heart rate, blood pressure (COLIN, Ohmeda), skin conductance, and respiration was performed using the SUEMPATHY device (SUEmpathie100, SUESS Medizin-Technik, Aue, Germany). The sampling frequency was 512 Hz for each channel. After 5 min of calibration and a test measurement, data acquisition commenced with the beginning of the experiment and lasted until the end of the presentation of all stimuli.

### Procedure

In total each participant received 24 stimuli; 12 control (4 sensory channels × 3 repetitions) + 12 disgust (4 sensory channels × 1 labeled disgust category + 4 sensory channels × 2 unlabeled disgust categories) in one out of 12 predefined orders. To attenuate the possibly aversive effect of repeated presentation of disgusting stimuli, each disgusting stimulus was followed by a control. However, only data from the first occurrence of each control stimulus was rated by the participants and only this one was analyzed with respect to autonomic measurements. Each participant received one of the disgust categories labeled (see Materials).

#### Stimuli were presented in the following way

The participants sat relaxed in a distance of about 70 cm in front of a monitor on which all instructions were presented. Prior to each stimulus participants read one of the following instructions “You are going to see/hear/touch/smell something”^2^. After this the screen went black for 10 s. For the next 10 s the stimuli were presented. Pictures were shown on the monitor, auditory stimuli were given through earphones. During the whole experiment, the participants stretched their left arm out with palms in a supine position. When tactile stimuli were presented, they were placed on the participants palm in order to allow the participants to touch these stimuli, without seeing them. Liquid odors were presented in small glass bottles filled with cotton pads. The experimenter put the opened bottles beneath the nose of the participants. The whole procedure was practiced with each participant before the experiment started. After 10 s of presentation, the stimuli were removed and a black screen was presented for 30 s. After this interval, participants were asked to judge the arousal and valence of the stimuli presented as well as the emotions evoked by the stimuli. The rating scales were presented via monitor and participants told their judgment to the experimenter who typed them in. There was no time limit to provide the subjective ratings. After ratings, the instruction for the next stimulus was presented.

Two repetitions took place about 2 and 4 weeks after the first experiment. Order of stimulus presentation remained unchanged.

### Analysis methods

**Figure 1:**
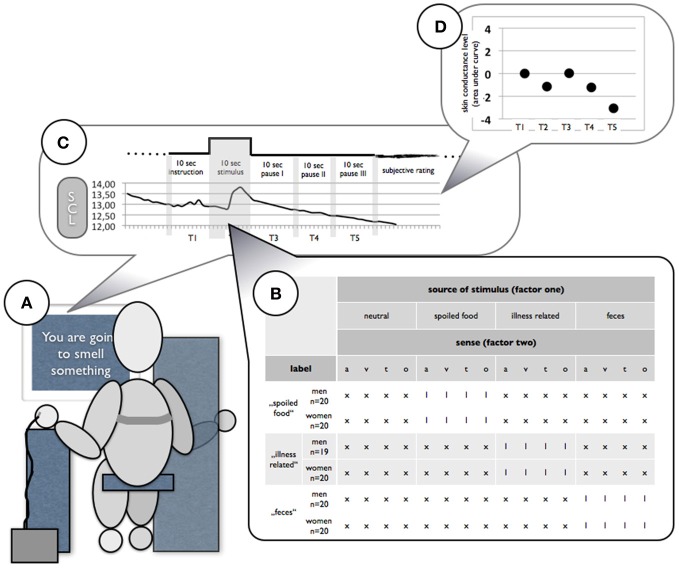
**Schematic visualization of the experimental setup. (A)** Participants sat in front of a monitor where instructions and pictures were presented. Auditory stimuli were presented via head set, olfactory were presented in opaque brown glass jars which were placed under the participant's nose and tactile stimuli were placed in the participant's supine hand without the participant seeing them. **(B)** Each participant received 16 different stimuli; one of the disgust categories was presented labeled for each participant. **(C)** During the whole experiment HR, SCL, and SBP were recorded. **(D)** After recording AUC was calculated to baseline for four 10-s intervals during and after stimulus presentation.

Off-line, a trained observer identified recordings with artifacts which were excluded from further analysis. Afterwards, data analysis was performed as area under the curve-analysis (AUC). The AUC measure was chosen to reduce large inter-individual variance as it is present in SCL amplitudes for instance. AUC analysis has been shown to be a better predictor of autonomic arousal then conventional analysis (Bach et al., 2010). The mean of data at T1 (10 s before the stimulus onset) served as baseline and AUC for data at T2 (1–10 s; during stimulus presentation), T3 (11–20 s, pause 1 after stimulus presentation), T4 (21–30 s, pause 2 after stimulus presentation), and T5 (31–40 s, pause 3 after stimulus presentation) was calculated according to this baseline (see Figure 1). We decided to split data into 10 s intervals in order to see potential autonomic correlation of both reactions: orienting and action preparing (Bradley et al., 2001). In result, a matrix was generated for each measurement (heart rate [HR], systolic blood pressure [SBP], and skin conductance level [SCL]), which encompassed 119 participants and AUC-data at T1, T2, T3, T4, T5 for each of 16 stimuli. For presentation purpose, the AUC was afterwards corrected by the factor 0.1, reflecting the mean increase or decrease in the 10s interval.

All data were analyzed using the SPSS 19 Software (SPSS Inc., Chicago, IL, USA). Data of the three different disgust categories were combined by averaging the responses to two unlabeled disgust stimuli of each sensory channel. Comparisons between evoked emotions and between control and disgusting stimuli were performed using ANOVA for repeated measurements with the within-subject-factors “disgust” (control vs. disgust) and “sense” (4). For autonomic data, timeline served as additional covariate representing the 4 measurement points after baseline.

The effect of label was analyzed for disgusting stimuli only, using “label” (2) as within-subject factor in the ANOVA. The effect of repeated stimulation was analyzed for those participants who took part in the experiment three times. Here, the first presentation was compared to the last one for all of the disgusting stimuli in the four different sensory channels. Only responses to unlabeled stimuli were analyzed. Level of significance was set at *p* = 0.05. Wherever appropriate, results are presented Bonferroni-corrected to minimize influence of multiple testing. This is indicated by “*p*_−corrN_,” with “*N*” indicating the number of comparisons for which the *P*-value is corrected.

## Results

### Ratings of disgust, valence, and arousal

**Figure 2:**
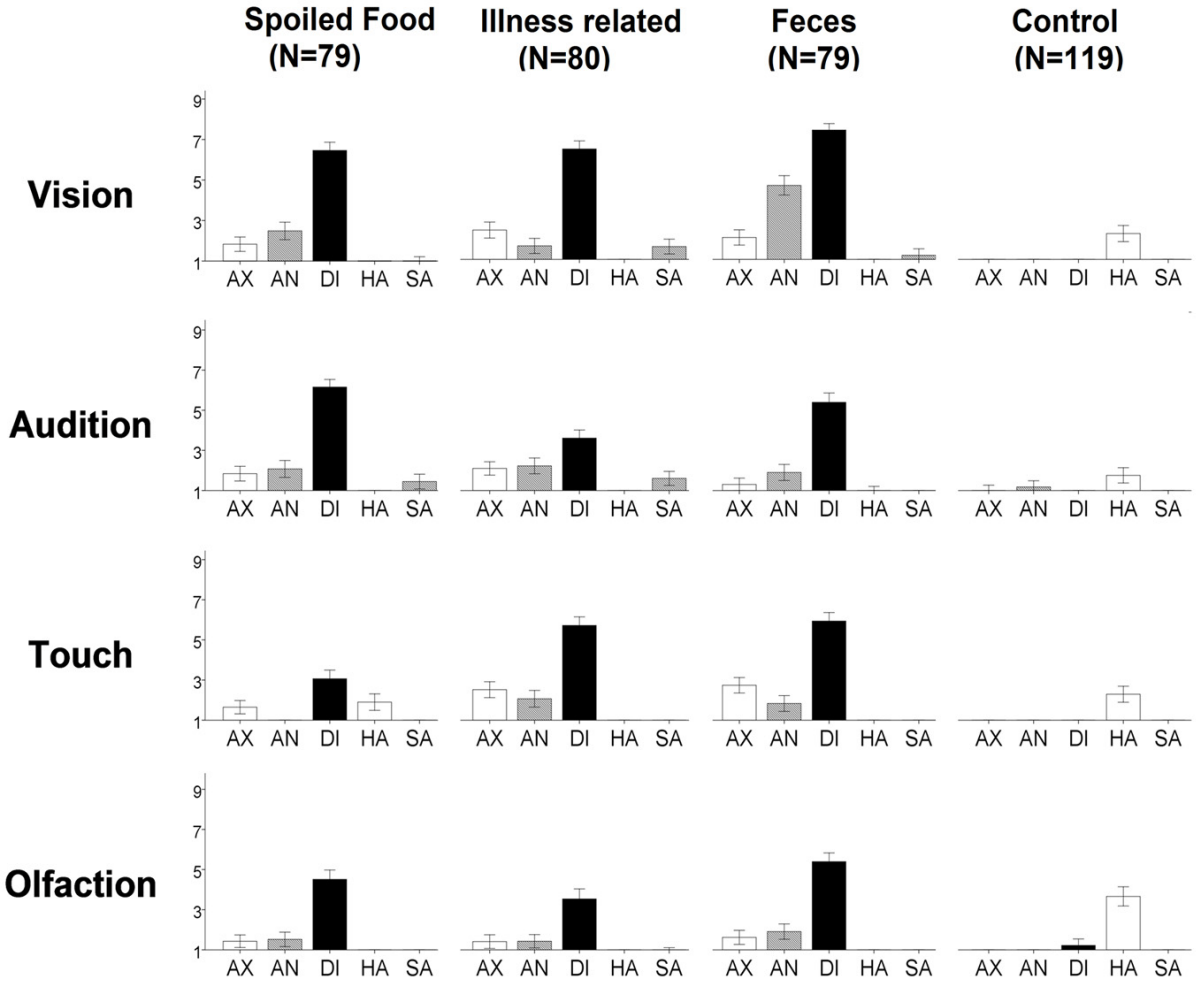
**Ratings of evoked emotion for each stimulus**. The y-axis represents the rating between 1 “not at all” and 9 “extremely strong” for the emotions anxiety (AX), anger (AN), disgust (DI), happiness (HA), and sadness (SA). Note: Error bars indicate 95% confidence interval.

Ratings of evoked basic emotion are visualized in Figure 2. For each of the disgusting stimuli, disgust was evoked more than in the matching control [*t*_(118)_ = 8.2–44.3; *p*_corr12_ < 0.01] and disgust was the emotion evoked most strongly [*t*_(118)_ = 6.1–35.6; *p*_corr4_ < 0.01], except for tactile-“spoiled food.” Here, disgust ratings were not significantly higher than ratings for happiness [*t*_(118)_ = 3.2; *p*_corr4_ = 0.06]. Together, this justifies merging the disgust categories for the following results section.

Comparison of the sensory channels revealed that seeing disgusting stimuli led to higher ratings of disgust than hearing, touching, or smelling them. This was the case for each of the three disgust categories [*t*_(118)_ = 3.1–13.1; *p*_corr9_ < 0.05], except for spoiled food where pictures did not get significantly higher ratings than sounds [*t*_(118)_ = 1.4; *p*_corr9_ > 0.05].

Ratings of hedonic valence and arousal are provided in Table 2. A main effect of disgust was revealed for valence and arousal, indicating that the disgusting stimuli were rated as less pleasant and more arousing compared to the controls. This was the case in all of the sensory channels [pleasantness: *F*_(1, 118)_ = 353.7–624.0, *p*_corr3_ < 0.01; arousal *F*_(1, 118)_ = 173.4–244.1; *p*_corr3_ < 0.01].

**Table 2:** **Ratings of hedonic valence and arousal of the disgusting and control stimuli applied through different senses (*N* = 119)**.

	**Vision**	**Audition**	**Touch**	**Olfaction**
Disgust	Valence: 2.8 ± 1.2	Valence: 3.6 ± 1.1	Valence: 4.7 ± 1.4	Valence: 4.0 ± 1.3
	Arousal: 5.1 ± 1.8	Arousal: 4.5 ± 1.8	Arousal: 4.0 ± 1.8	Arousal: 3.8 ± 1.7
Control	Valence: 6.2 ± 1.3	Valence: 5.5 ± 1.4	Valence: 6.3 ± 1.4	Valence: 6.5 ± 1.9
	Arousal: 1.9 ± 1.4	Arousal: 2.9 ± 1.9	Arousal: 2.4 ± 1.6	Arousal: 2.9 ± 1.9

Ratings are given on a 9-point scale with “1” meaning ”not at all” pleasant respective arousing and “9” meaning ”extremely” pleasant respective arousing. Only participants ratings for stimuli presented unlabeled are given in order to avoid possible label influences.

Focusing on the disgusting stimuli only, there was a significant main effect of the sensory channel, with visual stimuli being perceived as least pleasant [*F*_(3, 116)_ = 65.4; *p* < 0.01] and most arousing [*F*_(3, 116)_ = 23.2; *p* < 0.01].

### Reactions of the autonomic nervous system to disgusting stimuli

#### Comparison between disgusting and the matching control stimuli

**Figure 3:**
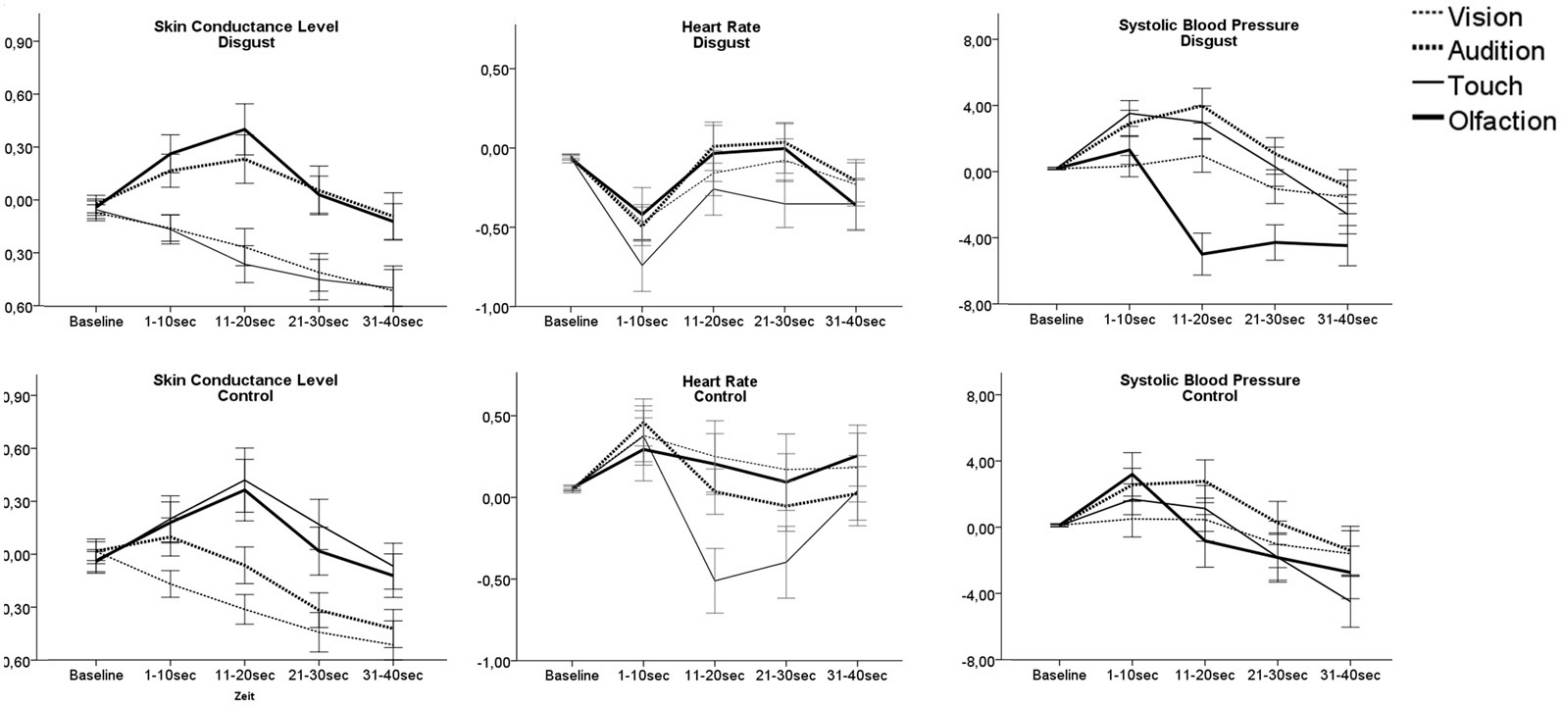
**Autonomic responses as AuC following presentation of disgusting and control stimuli**. Lines show mean response of the participants toward the three disgusting stimuli applied through each sense. Note: Only participants' response for stimuli presented unlabeled are presented in order to avoid possible label influences. Error bars indicate 95% confidence interval.

No significant main effect of sensory channel or disgust was revealed for HR but a significant interaction [*F*_(3, 113)_ = 3.6, *p* = 0.01, compare Table 3 and Figure 3]. *Post-hoc* test revealed a significant difference in the tactile channel. Here HR decreased in the disgust stimuli compared to the control [*F*_(3, 113)_ = 9.5, *p* < 0.01]. Similarly, for SCL a significant main effect of the sensory channel [*F*_(3, 113)_ = 10.0, *p* < 0.01] and a significant interaction [*F*_(3, 113)_ = 5.9, *p* < 0.01] was found. *Post-hoc* testing revealed that tactile disgusting stimuli led to significantly lower SCL compared to control [*F*_(1, 115)_ = 12.3, *p* < 0.01]. For SBP a significant main effect of the sensory channel [*F*_(3, 113)_ = 39.6, *p* < 0.01] and a significant interaction [*F*_(3, 113)_ = 22.9, *p* < 0.01] was found. *Post-hoc* testing revealed that tactile disgust stimuli led to enhanced SBP compared to control [*F*_(1, 115)_ = 21.6, *p* < 0.01] and auditory disgust stimuli tended to lead to enhanced SBP compared to control [*F*_(1, 115)_ = 3.5, *p* = 0.06]. Olfactory disgust stimuli on the other side led to reduced SBP compared to control [*F*_(1, 115)_ = 36.7, *p* < 0.01].

**Table 3:** **Autonomic measurements**.

**Presentation**	**Sense**	**Stimuli**	**SCL in μS**		**HR in bpm**		**SBP in mmHG**	
			**Mean**	**Standard deviation**	**Mean**	**Standard deviation**	**Mean**	**Standard deviation**
First presentation *N* = 116	Vision	Disgust	−0.17	0.61	−0.23	0.73	−2.8	44.6
		Control	−0.2	0.62	−0.25	1.13	−3.6	63.8
	Audition	Disgust	0.28	0.75	−0.16	0.76	**13.8**	**50.2**
		Control	0	0.64	−0.12	0.84	**8**	**64.3**
	Touch	Disgust	**−0.2**	**0.64**	**−0.43**	**0.88**	**8.1**	**55.3**
		Control	**0.37**	**0.88**	**0.12**	**1.13**	**−7.3**	**68.6**
	Olfaction	Disgust	0.34	0.71	−0.20	0.92	**−24.9**	**60.3**
		Control	0.3	0.83	−0.21	1.01	**−4.7**	**75.8**
Second presentation *N* = 47	Vision	Disgust	−0.39	0.72	−0.13	0.70	0.8	29
		Control	−0.12	0.43	−0.23	0.76	−1.8	42.4
	Audition	Disgust	0.13	0.71	0.03	0.68	4.4	26.2
		Control	−0.01	0.36	−0.31	0.71	5.2	47.1
	Touch	Disgust	−0.43	0.58	−0.49	0.84	1.8	36.1
		Control	0.15	0.64	0.18	0.93	−1.7	44.3
	Olfaction	Disgust	0.28	0.69	−0.16	0.85	−5.7	31.6
		Control	0.06	0.5	−0.28	0.94	−0.3	45.4
Third presentation *N* = 43	Vision	Disgust	−0.24	0.63	−0.10	0.66	0	24.4
		Control	−0.04	0.49	−0.09	1.08	4.4	51.8
	Audition	Disgust	0.04	0.65	−0.26	0.65	1.2	27.1
		Control	−0.03	0.43	−0.18	0.99	8.5	36.1
	Touch	Disgust	−0.35	0.66	−0.72	1.03	5	25.3
		Control	0.1	0.45	0.20	1.04	5.2	45.7
	Olfaction	Disgust	0.21	0.75	−0.09	0.70	−2.2	30.1
		Control	−0.02	0.54	−0.24	0.92	−3.3	37.3

In columns the changes of HR, SCL and SBP averaged over the four time points for are provided for the disgusting stimuli and the control stimulus applied through the sensory systems. For the disgust stimuli, only unlabeled presented stimuli are taken into account. Bold numbers indicate that there is a significant difference in autonomic response between the control and the disgusting stimuli in the sensory system (*p* < *0.05*).

#### Comparison of disgusting stimuli between sensory channels

Autonomic reaction patterns differed between the sensory channels for all measurements: SCL [*F*_(3, 113)_ = 98.2, *p* < 0.01], HR [*F*_(3, 113)_ = 10.4, *p* < 0.01], and SBP [*F*_(3, 113)_ = 80.4, *p* < 0.01]. *Post-hoc* testing showed that *SCL* responses toward olfactory and auditory stimuli differed from responses to tactile and visual stimuli (*p*_corr6_ < 0.01). Olfactory and auditory disgust stimuli led to a SCL peak 10–20 s after stimulus presentation, while tactile and visual stimuli lead to a slow decrease of SCL. For *HR* the decrease was strongest for tactile disgust stimuli (*p*_corr6_ < 0.01), while there was no significant difference between the other sensory stimuli.

For *SBP*, responses toward visual stimuli could be differentiated from others (*p*_corr6_ < 0.01) by leading to little SBP change, while disgusting stimuli applied through the auditory or tactile channel were followed by a slow and strong increase of SBP with compensatory decrease. Olfactory stimuli on the other side were followed by a biphasic reaction with short increase followed by a strong decrease of SBP, which was sign different from SBP response to other sensory evoked disgust (*p*_corr6_ < 0.01).

### Influence of label

**Figure 4:**
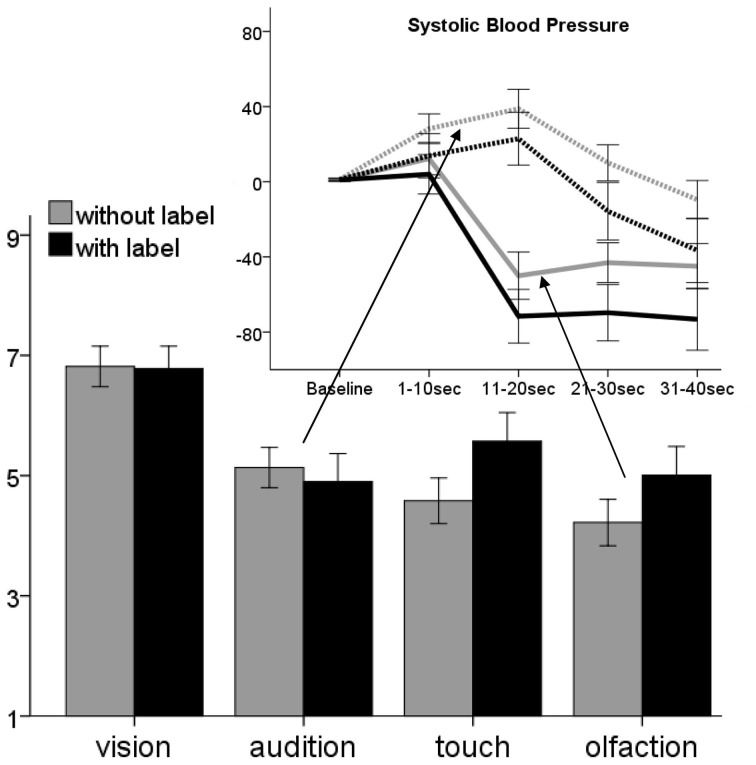
**Comparison of subjective disgust ratings toward stimuli presented with (black) and without (gray) labels**. Above the change of systolic blood pressure for auditory (dashed lines) and olfactory stimuli in the presence of label (black) is shown. Ratings are given on a 9-point scale, SBP in AuC intervals. Error bars indicate 95% confidence interval.

Labeling led to *enhanced disgust ratings of olfactory* [*t*_(117)_ = 2.6, *p*_corr4_ = 0.04, compare Figure 4] *and tactile* [*t*_(117)_ = 3.2, *p*_corr4_ = 0.01] *stimuli*. For visual and auditory stimuli no significant influence of labeling was found. Labeling significantly *enhanced HR deceleration in tactile* stimuli [*F*_(1, 115)_ = 6.9, *p*_corr4_ = 0.04, compare Table S1], *enhanced SBP decrease* following disgusting *olfactory* stimuli [*F*_(1, 115)_ = 26.8, *p*_corr4_ < 0.01] and diminished SBP reaction toward auditory stimuli [*F*_(1, 115)_ = 22.5, *p*_corr4_ < 0.01]. No significant influence of labeling was found for SCL response.

### Influence of repeated presentation

**Figure 5:**
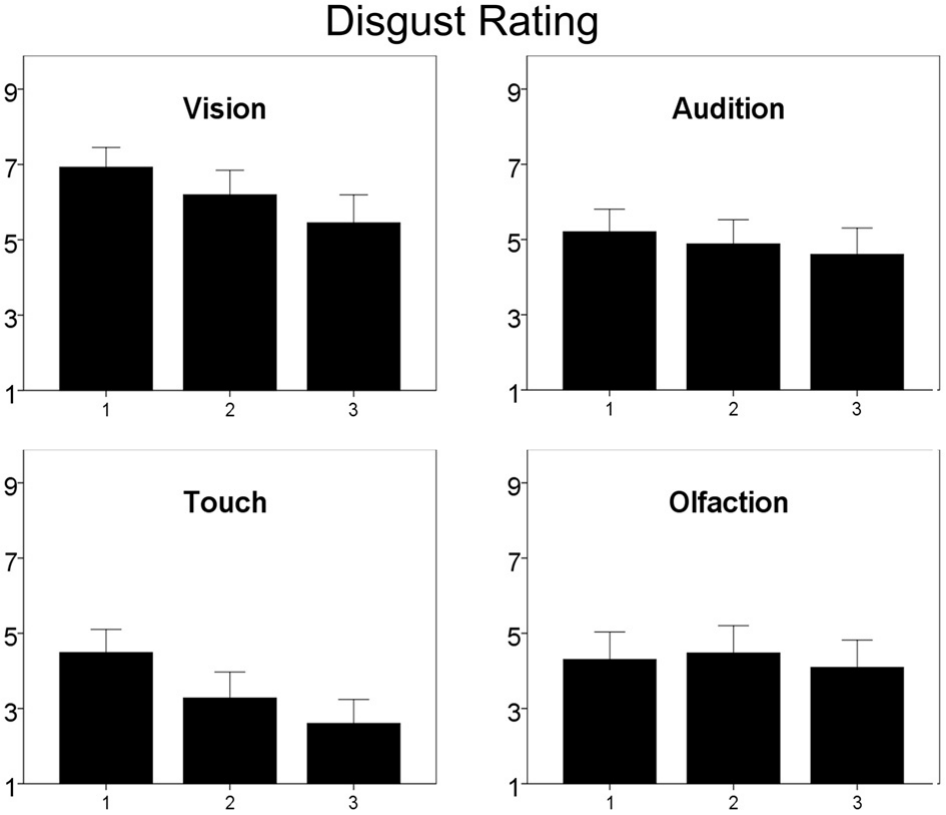
**Subjective ratings of disgust with repeated measurement**. Ratings are given on a 9-point scale. Higher values represent higher disgust ratings. Note: Only participants ratings for stimuli presented unlabeled are given in order to avoid possible label influences. Error bars indicate 95% confidence interval.

There was a significant main effect of repetition [*F*_(1, 42)_ = 20.2, *p* < 0.01, compare Figure 5] and a significant interaction between repetition and sensory channel [*F*_(3, 40)_ = 7.8, *p* < 0.01]. *Post-hoc* testing revealed a significant decrease of disgust ratings for visual [*F*_(1, 42)_ = 20.8, *p* < 0.01], tactile [*F*_(1, 42)_ = 31.6, *p* < 0.01], and auditory [*F*_(1, 42)_ = 3.8, *p* = 0.03], but not for olfactory stimuli.

For autonomic measurements either no effect or a diminished response was observed with repeated measurements (compare Table 3). In tendency, there was a main effect of repetition for HR [*F*_(3, 40)_ = 3.8, *p* = 0.05] and an interaction between repetition and the sensory channel [*F*_(3, 40)_ = 2.5, *p* = 0.06]. *Post-hoc* tests revealed a diminished reaction between the first and the third trial for the sense of touch [*F*_(1, 42)_ = 10.1, *p* < 0.01] but not for the other sensory channels.

There was a main effect of repetition for the SCL [*F*_(3, 40)_ = 31.2, *p* < 0.01], indicating that SCL reaction diminished with repeated presentation. However, there was no significant interaction between repetition and the sensory channel.

There was a main effect of repetition for the SBP [*F*_(3, 40)_ = 4.3, *p* = 0.04], and an interaction between repetition and the sensory channel [*F*_(3, 40)_ = 7.2, *p* < 0.01]. *Post-hoc* tests revealed a flattening of the SBP curve between the first and the third trial for the sense of olfaction [*F*_(1, 42)_ = 15.5, *p* < 0.01] and in tendency for audition [*F*_(1, 42)_ = 3.6, *p* = 0.06].

## Discussion

The study was designed to compare disgust reactions evoked through the visual, auditory, tactile, and olfactory sense. Confirming previous studies (Alaoui-Ismaili et al., 1997a; Collet et al., 1997; Bensafi et al., 2002; Hertenstein et al., 2009; Croy et al., 2011; Oum et al., 2011), the ratings show that disgust can be evoked through the visual, olfactory, and tactile channel. Furthermore, disgust could be evoked through the auditory channel using non-verbal information. To our knowledge this has not been shown before, though the finding is not very surprising.

We assumed that the sensory channel of presentation contributes to disgust reaction. Supporting this, autonomic reaction toward disgusting stimuli differed according to the channel of presentation. Labeling enhanced disgust reaction for olfactory and tactile, but not for visual and auditory stimuli. Furthermore, with repeated measurements participant's disgust rating diminished to all but olfactory applied stimuli. The results are discussed in detail below.

According to Bradley and colleagues autonomic reaction to an emotional cue is biphasic: The initial orienting reaction, indicated by deceleration of HR and increase of SCL, is replaced by an action tendency toward the stimulus (Bradley et al., 2001). We observed a HR deceleration within the first 10 s for all disgust stimuli, potentially reflecting an orienting reaction. An increase of SCL however, was only observed for disgusting auditory and olfactory stimuli.

After the initial orientation phase, autonomic reaction patterns differed between the senses. SCL decreased for visual and tactile stimuli but showed an increase for olfactory and auditory stimuli. SBP increased for auditory and tactile stimuli, but showed a strong decline for olfactory stimuli. Autonomic responses are highly dependent on context and relevant action tendencies (Van Diest et al., 2009) and the different the patterns observed may indicate different action tendencies.

*Olfaction* is strongly linked to food intake and plays a critical role in checking whether food is spoiled or edible. Consequently, people without a sense of smell report more often to have accidentally eaten spoiled food (Croy et al., 2012). In contrast to stimuli that are seen, heard or touched, olfactory stimuli have a relatively high probability to be in the mouth (via the retronasal pathway) or to be about to enter the body. Therefore, odors related to potential harmful substances may evoke a disgust reaction that prepares for vomiting. The HR reduction, indicating a vagal reaction, supports this hypothesis as well as the strong decrease of blood pressure, which has been found to be related to vomiting (Pusch et al., 2002).

For *visual* evoked disgust, autonomic response failed to show a clear effect compared to the controls. After the orienting reaction, visual evoked disgust reaction was mainly characterized by decrease of SCL. The weak autonomic reaction could indicate that visually evoked disgust (at least with the stimuli we used) does not initiate strong action tendencies. A similar slow SCL decrease was previously observed for the presentation of disgusted faces (Collet et al., 1997). In another study however, an increase of SCL in the first seconds following presentation of disgusting pictures is reported (Bradley et al., 2001). An explanation might be that the authors analyzed the maximum SCL amplitude in a given time interval compared to baseline. We used a more conservative approach by analyzing AUC. Interestingly, the relatively weak effects of visual presented disgust stimuli were accompanied by the highest disgust ratings. We assume that visual stimuli evoke stronger memory traces than tactile or olfactory stimuli, because they can be categorized easily (Baddeley and Hitch, 1974). This information contributes to emotional experience (Bradley, 2000) and may enhance disgust ratings.

Autonomic disgust reaction evoked by *auditory* stimuli was characterized by a significant, but relatively low, increase of SCL and SBP, indicating sympathetic activation. This prepares the body for fast reaction and could indicate a weak fight and flight action tendency.

The *tactile* channel has to be interpreted with caution for two reasons: The stimulus characteristics were not obvious at once but changed over time, while the participants touched the object. This may influence experience, as indicated by the ratings: Although the participants experienced tactile objects as clearly disgusting, they were not rated very unpleasant. Furthermore, the autonomic measures of tactile stimuli were influenced by the participants moving the fingers of the non-attached side. Those circumstances might result in altered autonomic reaction and enhanced orienting as indicated by the strong decrease of HR. The *effect of labeling* supports the differential impact of the senses. Labeling increased disgust ratings and autonomic reaction toward disgusting odors and tactile cues, but not for auditory and visual ones. Labeling adds contextual information. However, for visual and even auditory stimuli labeling presumably did not add more information than that already retrieved from memory. For olfactory and tactile stimuli on the other hand, labeling altered the response. This is in line with previous studies for the sense of smell (De Araujo et al., 2005; Bensafi et al., 2007, 2012) and an interesting finding for the sense of touch.

In accordance with our hypothesis, disgust reaction for *repeated presentation* also differed between the sensory channels: For all but olfactory stimuli disgust ratings decreased. As olfactory stimuli are hard to identify (Jonsson and Olsson, 2003), recognition is difficult. That may explain that emotional response did not decrease with repeated presentation. We hypothesized a similar effect for auditory and tactile cues. However, the effect of labeling suggests that auditory stimuli evoked a lot of context information. For tactile information on the other hand, carefully touching the objects for 10 s could lead to enlarged encoding, which would make recognition easier. The autonomic response toward disgusting stimuli either decreased over time or remained unchanged. No enhanced response was observed with repeated measurement, suggesting that there was no sensitization toward disgusting stimuli.

We are aware of several *limitations* of the study. First, the stimuli differed in intensity and hedonic value: Visual applied stimuli were rated as most unpleasant and arousing. This could either indicate a bias in the choice of stimuli or disgust is in fact more intense if evoked through the sense of vision compared to other senses. Although being rated more arousing, unpleasant and disgusting, visual disgust stimuli did not evoke more anger, sadness, happiness, or anxiety than disgust stimuli applied through the other senses. In order to clarify the influence on autonomic measurements, one has to take care to match stimuli in intensity. This can be done by reducing intensity of the visual stimuli, for instance by reducing contour or color of the pictures. However, ecological validity should be preserved. For the autonomic results enhanced intensity in the visual stimuli should rather over-than underestimate the effect, but still there was no significant difference in autonomic measurements between the visual control and disgust stimuli.

Second, all of the control stimuli were rated rather positive. This may reflect an inter class bias (Hoyt, 2000), meaning that the disgusting stimuli enhanced the contrast to the control ones and therefore the control stimuli were rated more positive. Third, the autonomic measurements used were not specific for disgust reaction. A facial EMG at the levator nasi muscle could add useful information. And fourth, in order to keep the already complex design as simple as possible, we did not include a control emotion. This might be a problem, as the participants may at some point of the experiment be aware that half of the stimuli are rather disgusting. That could bias their answers toward the disgust category. Future studies should investigate whether threatening or joy evoking stimuli, for instance, are perceived in another way when heard, seen, touched, or smelled.

For disgust, we argue that the sensory channel of presentation contributes to the emotional experience. This might also integrate the controversial findings of autonomic measurements on disgust (Kreibig, 2010). Therefore, research on emotions should pay more attention on the sensory channel, through which emotions are evoked.

### Conflict of interest statement

The authors declare that the research was conducted in the absence of any commercial or financial relationships that could be construed as a potential conflict of interest.

## Footnotes

^1^We do not examine the sense of gustation, because the pure sense of gustation only involves the qualities sour, sweet, salty, bitter, and umami. Although bitterness might provide an excellent disgust induction technique, it is not possible to present the aforementioned disgust categories purely through the gustatory channel. In order to keep the design comparable across the senses, we decided not to examine taste.

^2^In case of labeling the instruction was modified. For instance in the disgust category “spoiled food” the label was modified to: (“you are going to see a picture of rotten food/are going to hear a person who ate something spoiled/going to smell rotten food/going to touch rotten food”). For category “illness related” the instruction was changed so that the participants were warned about a “sick person,” in feces category they were warned about “feces.”

## Supplementary material

Table S1 **Effect of labeling on autonomic measurement**.
