# Supplementary material for: The sensory channel of presentation alters subjective ratings and autonomic responses toward disgusting stimuli—Blood pressure, heart rate and skin conductance in response to visual, auditory, haptic and olfactory presented disgusting stimuli
Source: Front Hum Neurosci. 2013 Sep 3;7:510. doi: 10.3389/fnhum.2013.00510 (PMC3759797; doi:10.3389/fnhum.2013.00510)
Supplement: Table S1 — Effect of labeling on autonomic measurement. [file DataSheet1.PDF]

The sensory channel of presentation alters subjective ratings and autonomic responses towards disgusting stimuli

- Blood pressure, heart rate and skin conductance in response to visual, auditory, haptic and olfactory presented disgusting stimuli-

Croy, I [1,3], Laqua, K[1], Süß, F [2], Joraschky [3], P, Ziemssen [4], T & Hummel, T [1]

[1] Smell and Taste Clinic, Department of Otorhinolaryngology, University of Dresden Medical School, Fetscherstr 74, 01307 Dresden, Germany

[2] Department of Occupational and Social Medicine, University of Dresden Medical School, Fetscherstr 74, 01307 Dresden, Germany

[3] Department of Psychosomatic Medicine, University of Dresden Medical School, Fetscherstr 74, 01307 Dresden, Germany

[4] Center of Clinical Neuroscience, Neurological University Clinic, University of Dresden Medical School, Fetscherstr 74, 01307 Dresden, Germany

Table S1: Effect of labeling on autonomic measurement. In rows changes for HR, SCL and SBP are provided for stimuli presented with or without label. In columns the time the change refers to is presented. * indicates that there is a significant difference in autonomic response between labeled and unlabeled disgusting stimuli in the sensory system (p_{corr}<0.001).

			T2<br>During stimulus presentation<br>0-10sec		T3<br>pause 1<br>11-20sec		T4<br>pause 2<br>21-30 sec		T5<br>pause 3<br>31-40sec	
			Mean	SD	Mean	SD	Mean	SD	Mean	SD
**HR in bpm**	Vision	with label	-0.45	-0.47	-0.17	-0.49	-0.20	-0.54	-0.29	-0.50
		without label	-0.47	-0.50	-0.16	-0.63	-0.08	-0.57	-0.22	-0.62
	Audition	with label	-0.39	-0.50	0.02	-0.61	0.08	-0.60	-0.12	-0.60
		without label	-0.50	-0.53	0.01	-0.70	0.04	-0.56	-0.21	-0.61
	Touch*	with label	-0.90	-0.56	-0.54	-0.50	-0.42	-0.75	-0.52	-0.73
		without label	-0.74	-0.72	-0.26	-0.66	-0.35	-0.66	-0.35	-0.75
	Olfaction	with label	-0.28	-0.64	-0.10	-0.53	-0.18	-0.48	-0.40	-0.66
		without label	-0.42	-0.76	-0.04	-0.79	0.00	-0.65	-0.35	-0.67
**SCL in µS**	Vision	with label	0	0.22	-0.19	0.29	-0.27	0.37	-0.36	0.51
		without label	0.03	0.36	-0.1	0.49	-0.24	0.49	-0.35	0.57
	Audition	with label	0.36	0.37	0.45	0.55	0.23	0.65	0.06	0.7
		without label	0.38	0.42	0.44	0.64	0.25	0.65	0.09	0.69
	Touch	with label	0.07	0.37	-0.18	0.46	-0.3	0.6	-0.36	0.64
		without label	0.01	0.34	-0.2	0.49	-0.29	0.55	-0.34	0.56
	Olfaction	with label	0.47	0.41	0.55	0.57	0.24	0.54	0.04	0.6
		without label	0.46	0.53	0.61	0.69	0.22	0.51	0.06	0.5
**SBP in mmHG**	Vision	with label	0.34	3.03	-0.29	3.28	-2.1	3.6	-3.28	5.34
		without label	-0.14	3.09	0.38	3.86	-1.12	4.12	-0.86	4.35
	Audition*	with label	1.29	3.82	2.31	3.87	-1.38	4.66	-3.76	6.66
		without label	2.3	3.64	2.89	3.71	0.9	4.13	0.14	5.12
	Touch	with label	3.52	3.58	1.84	3.97	-0.77	4.25	-3.04	4.93
		without label	2.11	3.43	2.7	4.16	0.61	4.47	-1.61	4.41
	Olfaction*	with label	0.43	3.2	-7.02	4.18	-6.94	4.86	-7.34	4.32
		without label	0.06	3.34	-4.2	4.97	-3.13	4.29	-4.54	5.1
